# *Helicobacter pylori iceA*, Clinical Outcomes, and Correlation with *cagA*: A Meta-Analysis

**DOI:** 10.1371/journal.pone.0030354

**Published:** 2012-01-18

**Authors:** Seiji Shiota, Masahide Watada, Osamu Matsunari, Shun Iwatani, Rumiko Suzuki, Yoshio Yamaoka

**Affiliations:** 1 Department of Environmental and Preventive Medicine, Oita University Faculty of Medicine, Hasama-machi, Yufu-City, Oita, Japan; 2 Department of General Medicine, Oita University Faculty of Medicine, Hasama-machi, Yufu-City, Oita, Japan; 3 Department of Medicine-Gastroenterology, Baylor College of Medicine and Michael E. DeBakey Veterans Affairs Medical Center, Houston, Texas, United States of America; National Cancer Center, Japan

## Abstract

**Background:**

Although the *iceA* (induced by contact with epithelium) allelic types of *Helicobacter pylori* have been reported to be associated with peptic ulcer, the importance of *iceA* on clinical outcomes based on subsequent studies is controversial. The aim of this study was to estimate the magnitude of the risk for clinical outcomes associated with *iceA*.

**Methods:**

A literature search was performed using the PubMed and EMBASE databases for articles published through April 2011. Published case-control studies examining the relationship between *iceA* and clinical outcomes (gastritis, peptic ulcer, including gastric ulcer and duodenal ulcer, and gastric cancer) were included.

**Results:**

Fifty studies with a total of 5,357 patients were identified in the search. Infection with *iceA1*-positive *H. pylori* increased the overall risk for peptic ulcer by 1.26-fold (95% confidence interval [CI], 1.09–1.45). However, the test for heterogeneity was significant among these studies. Sensitivity analysis showed that the presence of *iceA1* was significantly associated with peptic ulcer (odds ratio [OR] = 1.25, 95% CI = 1.08–1.44). The presence of *iceA2* was inversely associated with peptic ulcer (OR = 0.76, 95% CI = 0.65–0.89). The presence of *iceA* was not associated with gastric cancer. Most studies examined the *cagA* status; however, only 15 studies examined the correlation and only 2 showed a positive correlation between the presence of *cagA* and *iceA1*.

**Conclusion:**

Our meta-analysis confirmed the importance of the presence of *iceA* for peptic ulcer, although the significance was marginal.

## Introduction

*Helicobacter pylori* infection is now accepted as the major cause of chronic gastritis. Several epidemiological studies have shown that *H. pylori* infection is also linked to severe gastritis-associated diseases, including peptic ulcer disease (PUD) and gastric cancer (GC) [1]. In 1994, the International Agency for Research on Cancer categorized *H. pylori* infection as a group I carcinogen (definite carcinogen) [2]. The infection remains latent in the majority of infected patients, with only approximately 20% of infected individuals developing severe diseases. In addition to host, environmental, and dietary factors, another possible reason for the various outcomes of *H. pylori* infection relates to differences in the virulence of *H. pylori* strains. Several *H. pylori* virulence factors have been reported to be associated with peptic ulcer and GC, including *cagA*, *vacA*, *babA*, and *oipA* [1], [3]–[6]. We recently reported the importance of the duodenal ulcer-promoting gene (*dupA*) for developing duodenal ulcer (DU) in a meta-analysis model [7].

An initial series of studies showed that *iceA* (induced by contact with epithelium) has 2 main allelic variants, *iceA1* and *iceA2* [8], [9]. *iceA1* demonstrated sequence homology with a gene from *Neisseria lactamica*, *nla*IIIR, which encodes a CTAG-specific restriction endonuclease [9]. On the other hand, *iceA2* has no homology to known genes and the function of the *iceA2* product remains unclear. van Doorn *et al.* reported that the *iceA* allelic type was independent of the *cagA* and *vacA* status, and there was a significant association between the presence of the *iceA1* allele and PUD [8]. The expression of *iceA1* was upregulated on contact between *H. pylori* and human epithelial cells, and the *iceA1* genotype was linked with enhanced mucosal interleukin (IL)-8 expression and acute antral inflammation [9], [10].

However, the role of *iceA* was controversial subsequently since several studies were not able to reproduce the observation in other populations, including Japanese [11]–[15]. Such discrepant results between the *iceA* allelic type and clinical outcomes could be explained by the genetic heterogeneity or differences in the geographic location, which were previously reported for other virulence genes [16]. So far, there is no report on the significance of *iceA* using meta-analysis. In this study, we aimed to perform a meta-analysis to examine the relationship between the *iceA* allelic type and clinical outcomes.

## Materials and Methods

A literature search was performed using the PubMed and EMBASE databases for articles published through April 2011. The following text words were used: 1) *iceA, iceA1*, or *iceA2* and 2) *pylori* or *Helicobacter*. We did not include abstracts alone or unpublished articles.

### Inclusion Criteria

The following criteria were applied to select fully published case-control studies examining the relationship between the *iceA* type and clinical outcomes (gastritis, PUD, gastric ulcer [GU], DU, and GC) in adult populations: the presence of *iceA* (*iceA1* or *iceA2*) was examined by polymerase chain reaction (PCR) and original articles were published in English. Studies were excluded if no raw data were presented. When it appeared that the same subjects were presented in multiple reports, the earliest article was selected. All potentially relevant articles were reviewed independently by 2 investigators (S.S and Y.Y) and disagreement was resolved by discussion.

### Exclusion Criteria

Studies were excluded if no raw data were presented or if no control groups were included. In addition, *in vitro* studies and studies conducted in child populations were also excluded.

### Data Extraction

Data were extracted independently from each study by the investigators and entered into a computerized database. The information retrieved covered countries where the study was performed, characteristics of cases and controls, the method for detection of *iceA*, the number of subjects, and the *iceA* status according to clinical outcomes. Two studies examined the prevalence of *iceA* in several countries [6], [11]; thus, the data of each country were entered in separate sheets as an independent study.

### Statistical analysis

Summary odds ratios (ORs) and 95% confidence intervals (CIs) were calculated from raw data. The Mantel-Haenszel method was used to test for statistical heterogeneity. When statistical heterogeneity was noted, the proportion of the total heterogeneity variance was calculated from each study using a fixed-effects model to guide the search for sources of methodologically and clinically important variables. To exclude any possible influence of a single study, we performed a sensitivity analysis to evaluate whether the exclusion of any single study substantially altered the magnitude or statistical result of the summary estimate. Publication bias was assessed by funnel plots and regression test by Egger *et al* [17]. A P value of <0.05 was considered as statistically significant. All analyses were performed using Comprehensive Meta-Analysis software (version 2, Biostat, Englewood, NJ).

## Results

The literature searches generated 140 potentially relevant citations. Of these, 93 articles were excluded. The main reasons for exclusion were that the articles were review articles, not case-control studies, conducted in children, and *in vitro* studies. Therefore, 46 articles met the inclusion criteria (Figure S1 and Table S1). Two articles included several countries (4 in Yamaoka *et al.* [11] and 2 in Yamaoka *et al.* [6]). For these studies, data from different countries in the same article were considered as separate studies (data); therefore, 50 studies with a total of 5,357 patients met the inclusion criteria. Among these studies, 28 were from Asian [11]–[15], [18]–[39], 19 from Western [6], [8], [9], [11], [40]–[52], and 3 from African countries [53]–[55]. An age- and sex-matched case-control study was conducted in only 1 report [47]. Although 46 articles showed a difference in the *iceA* type between gastritis and PUD, 14 articles did not show the distribution of GU and DU [12], [18]–[20], [24], [28], [32], [36], [39], [43], [48], [52], [53], [55]. Four studies examined the prevalence of *iceA* types in gastritis and GC, but not in PUD [13], [26], [44], [51]. Six studies examined only the prevalence of *iceA1* but not *iceA2* [6], [13], [20], [22], [41]. The prevalence of mixed infection by *iceA1* and *iceA2* was also different in each study; it ranged from 1.9% [14] to 36.7% [40]. Overall, the rate of mixed infection was 13.9%. Although strains from mixed infections were excluded in several studies [23], [29], [43], [46], [49], most studies included these mixed genotypes as total denominator. In this study, *iceA1*-positive means only *iceA1*-positive cases, but not *iceA1*- and *iceA2*-positive. Likewise, *iceA2*-positive means only *iceA2*-positive cases, but not *iceA1*- and *iceA2*-positive.

### Association between the *iceA1* status and clinical outcomes

**Figure 1:**
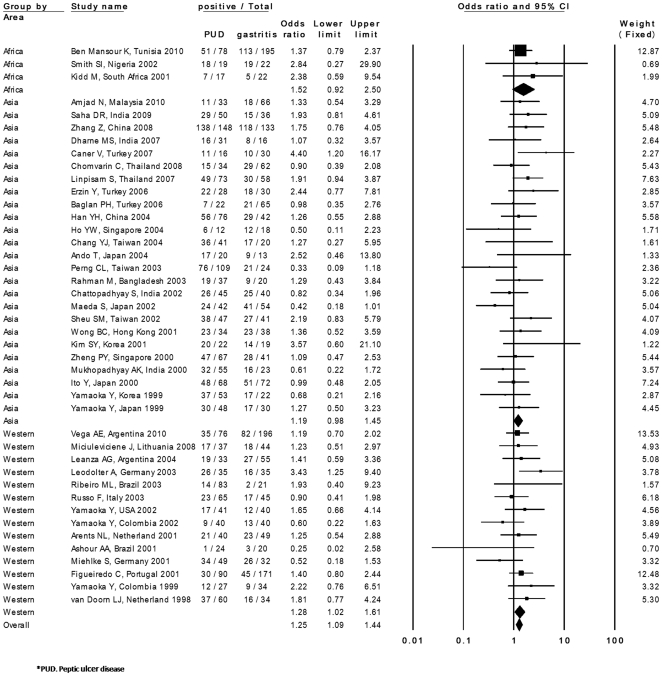
Results of the meta-analysis for the risk of peptic ulcer in *iceA1*-positive *H. pylori* infections. Odds ratios (ORs) and their 95% confidence intervals (CI) in summary and for each study are presented using a fixed-effect model. PUD: peptic ulcer disease.

The prevalence of *iceA1* in PUD patients was examined in 46 studies from 24 countries. The prevalence of *iceA1* ranged from 0.0% to 100.0% in PUD patients and from 4.3% to 100.0% in controls. Among 46 studies, a significantly higher prevalence of *iceA1* in PUD compared with controls was found in 4 studies [9], [32], [35], [47]. Only 1 study showed a significantly lower prevalence of *iceA1* in PUD patients compared with controls [11]. The overall prevalence of *iceA1* was 59.3% (1,264 of 2,131) in PUD patients and 51.0% (1,260 of 2,470) in controls. The summary OR in the fixed-effects model was 1.26 (95% CI, 1.09–1.45). However, the test for heterogeneity was significant among these studies (I squared  =  35.5, P = 0.01), suggesting the existence of either methodological or clinical heterogeneity. By exploring the sources of heterogeneity, we found that the studies by Peek *et al.* [9] and Momenah *et al.* [32] showed larger differences in the prevalence of *iceA1* compared with other studies. They reported a quite low prevalence of *iceA1* in controls (4.3% and 5.4%, respectively) compared with PUD patients (47.6% and 100%, respectively). In addition, the study by Yamaoka *et al.* in the United States showed a lower prevalence of *iceA1*, especially in PUD patients (0.0%) [11]. The study by Gatti *et al.* showed 100% prevalence in controls [49]. Sensitivity analysis excluding these 4 studies showed a similar OR (1.25; 95% CI, 1.08–1.44) and the test for heterogeneity was no longer statistically significant (I squared  =  4.5, P = 0.38) (Figure 1). Publication bias did not exist (intercept  =  0.17; P = 0.36).

Subgroup analysis by 2 areas (Asian or Western countries) was also performed. The prevalence of *iceA1* was 68.9% (840 of 1,218) in PUD patients and 58.1% (676 of 1,163) in controls in Asian countries. It was 43.5% (348 of 799) in PUD patients and 41.8% (447 of 1,068) in controls in Western countries. The summary OR was 1.19 (95% CI, 0.97–1.45) in Asian countries and 1.28 (95% CI, 1.02–1.61) in Western countries. Subgroup analysis was also performed according to the distribution of DU and GU. The prevalence of *iceA1* in DU patients was examined in 32 studies. When 4 studies [9], [11], [32], [49] of larger effect size were excluded to limit heterogeneity, *iceA1* was significantly associated with DU compared with controls (OR = 1.35, 95% CI = 1.11–1.63) (Figure S2). This finding was significant in Asian countries (OR = 1.38, 95% CI = 1.06–1.79). The prevalence of *iceA1* was examined in GU patients from 15 studies. One study included only 1 patient with GU [9]; therefore, this study was excluded from the statistical analysis. There was no association between *iceA1* and GU compared with controls (OR = 0.96, 95% CI = 0.69–1.35) (Figure S3). The prevalence of *iceA1* was examined in GC patients from 23 studies. One study included only 1 patient with GC [9]; therefore, this study was excluded from the statistical analysis. There was no association between *iceA1* and GC compared with controls (OR = 1.08, 95% CI = 0.86–1.37) (Figure S4).

### Association between the *iceA2* status and clinical outcomes

**Figure 2:**
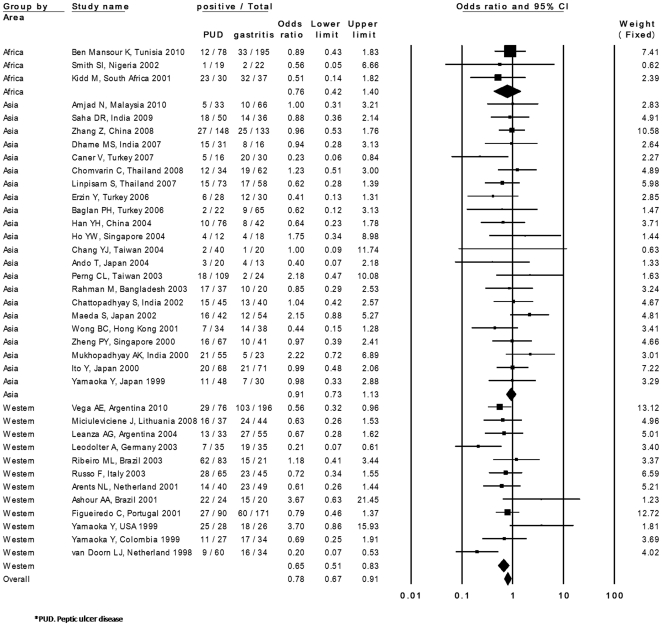
Results of the meta-analysis for the risk of peptic ulcer in *iceA2*-positive *H. pylori* infections.

The prevalence of *iceA2* was examined in PUD patients from 41 studies (24 countries). The prevalence of *iceA2* ranged from 0.0% to 91.7% in PUD patients and from 0.0% to 95.7% in controls. Among the 41 studies, a significantly lower prevalence of *iceA2* in PUD patients compared with controls was found in 6 studies [8], [9], [32], [35], [47], [52]. The overall prevalence of *iceA2* was 30.1% (585 of 1,944) in PUD patients and 37.2% (841 of 2,261) in controls. The summary OR in the fixed-effects model was 0.76 (95% CI, 0.65–0.89). However, the test for heterogeneity was significant among these studies (I squared  =  40.7, P = 0.004). The studies by Peek *et al.* [9] and Momenah *et al.* [32] showed larger differences in the prevalence of *iceA2* compared with other studies. They reported a quiet high prevalence of *iceA2* in controls (95.7% and 94.6%, respectively) compared with PUD patients (47.6% and 0.0%, respectively). In addition, the study by Yamaoka *et al.* in Korea showed a lower prevalence of *iceA2*, especially in gastritis patients (4.5%) [11]. The study by Gatti *et al.* showed 0.0% prevalence in controls [49]. Sensitivity analysis excluding these 4 studies showed a similar OR (0.78; 95% CI, 0.66–0.91) and the test for heterogeneity was no longer statistically significant (I squared  =  19.9, P = 0.14) (Figure 2). Publication bias did not exist (intercept, 0.35; P = 0.27).

Subgroup analysis by 2 areas (Asian or Western countries) was also performed. The prevalence of *iceA2* was 23.4% (269 of 1,148) in PUD patients and 30.2% (318 of 1,051) in controls in Asian countries. It was 41.8% (280 of 669) in PUD patients and 47.7% (456 of 956) in controls in Western countries. The summary OR was 0.91 (95% CI, 0.73–1.13) in Asian countries and 0.64 (95% CI, 0.50–0.82) in Western countries. Subgroup analysis was performed according to the distribution of DU and GU. The prevalence of *iceA2* in DU patients was examined in 27 studies. When 4 studies [9], [11], [32], [49] of larger effect size were excluded to limit heterogeneity, *iceA2* was significantly associated with DU compared with controls (OR = 0.76, 95% CI = 0.60- 0.92) (Figure S5). This finding was significant in Western countries (OR = 0.66, 95% CI = 0.48–0.91). The prevalence of *iceA2* was examined in GU patients from 13 studies. There was no association between *iceA2* and GU compared with controls (OR = 1.01, 95% CI = 0.69–1.46) (Figure S6). The prevalence of *iceA2* was examined in GC patients from 18 studies. One study included only 1 patient with GC [9]; therefore, this study was excluded from the statistical analysis. There was no association between *iceA2* and GC compared with controls (OR = 0.84, 95% CI = 064–1.11) (Figure S7). Subgroup analysis according to the area showed that the OR was 1.10 (95% CI, 0.72–1.67) in Asian countries and 0.70 (95% CI, 0.49–1.00) in Western countries.

### Difference in prevalence in Asian and Western countries

The overall prevalence of *iceA1* was 64.6% (1,791 of 2,771) in Asian countries and 42.1% (935 of 2,218) in Western countries. It was significantly higher in Asian countries than in Western countries (P<0.0001). On the other hand, the prevalence of *iceA2* was 25.8% (651 of 2,522) in Asian countries and 45.1% (844 of 1,871) in Western countries. It was significantly higher in Western countries than in Asian countries (P<0.0001).

### Correlation between *cagA* and *iceA*

Fifteen studies examined the correlation between the *cagA* and *iceA* status. Only 1 study showed a significant positive association between *iceA1* and the presence of *cagA* [41]. One study showed a positive trend [9]. Another study showed a significant positive association between *iceA2* and the presence of *cagA* [42]. Twelve studies showed no association between *iceA1* and *cagA* status [8], [11], [19]–[21], [31], [36], [49], [52].

## Discussion

Our present meta-analysis shows that the presence of *iceA1* was significantly associated with PUD. Although several studies failed to show a positive association between the *iceA* status and clinical outcomes, this meta-analysis supported the original report from 1998 [9]. However, this association was not very strong.

The mechanism of the development of PUD induced by *iceA* remains unclear. *iceA1* demonstrated sequence homology with a gene from *Neisseria lactamica*, *nla*IIIR, which encodes a CTAG-specific restriction endonuclease [9]. However, studies on the genetic organization of the *iceA* locus indicated that a full-length *nla*IIIR-like open reading frame has only been observed in 10 (20.4%) of 49 *iceA1 H. pylori* strains and only full-length *iceA1* was a functional endonuclease gene [10]. These data indicate that mutations in *iceA1* are common, resulting in protein products with poor or no endonuclease activity. It remains to be determined whether *iceA1* plays a role other than encoding an *nla*III-like endonuclease or not.

Most isolates with an *iceA2* allele could be divided into 2 types according to the presence of repeated sequences of 105 nucleotides and the size of the PCR products (229 bp for *iceA2-*1 or 334 bp for *iceA2*-2) [8]. Ashour *et al.* reported that no association was observed between the size of the *iceA2* amplicon and diseases [42]. On the other hand, Kidd *et al.* reported that the 334-bp *iceA2* amplicon was more prevalent in strains from patients with PUD [53]. These changes may translate into differential binding or function of the protein. The function of *iceA2* remains unclear.

The prevalence of mixed infection by *iceA1* and *iceA2* was also different in each study. The strains of mixed infections were excluded in several studies [23], [29], [43], [46], [49]. Figueiredo *et al.* reported that 36.7% of strains were positive for both *iceA1* and *iceA2*, and 53.8% of these strains also contained multiple *vacA* genotypes [40]. In our previous study, the rate of both *iceA1* and *iceA2* positivity was significantly lower in the United States than in Korea, Japan, and Columbia (4.3% vs. 20.0, 17.0, and 22.4%, respectively) [11]. Multiple genotypes indicate the presence of multiple strains because there is no full-sequenced strain containing both *iceA1* and *iceA2* genes in Genbank (data not shown). It may be speculated that more than 1 strain may be acquired in childhood, especially in countries with a high prevalence of *H. pylori*. Mixed infection by more than 1 strain in the same individual may reflect the capacity of *H. pylori* to evolve genetic variations during long-term colonization from childhood [56]. The high prevalence of mixed *iceA*-type strains may obscure any potential relationship between the allele and clinical outcomes.

Fourteen studies combined DU with GU as PUD. However, DU and GU are linked to entirely different patterns of gastric inflammation, such that it would seem they should be examined separately [6]. Tham *et al.* indicated that in patients with *H. pylori* infection, those with DU have a higher degree of acute and chronic inflammation in the gastric antrum and higher *H. pylori* density than those with GU [57], which illustrates the different pathogenic processes of DU and GU. Therefore, a study to examine the roles of virulence factors needs to be conducted according to DU and GU, respectively. Furthermore, we should pay attention to the fact that patients with only gastritis at the time of endoscopy may develop ulcer diseases later in life and, therefore, may have been misclassified in the present study [8].

The association between the *iceA* and *cagA* status remains unclear. Several virulence factors of *H. pylori* correlated with the presence of *cagA* [6], [58], [59]. In this study, we found that 15 studies examined the association between the *iceA* and *cagA* status. As a result, most of them showed no association, indicating that *iceA1* might be a risk factor for PUD, independent of *cagA*. In addition, our recent meta-analysis showed that *dupA*, which induces DU and has a suppressive action on GC, was significantly associated with DU [7]. The association between *dupA* and *iceA* has not been clarified yet; however, 1 study showed that there was no association [37]. To confirm the significance of *iceA*, it is better to perform a multivariate analysis adjusted for the *cagA* status and other risk factors for peptic ulcer. However, unfortunately, we could not obtain the raw data from each study. It is difficult to perform a multivariate analysis adjusted for these factors without the raw data. In addition, most studies did not consider other risk factors in their papers. Further study is necessary to examine which factors are true virulence factors and which are just confounding factors. However, it might be better to hypothesize that these factors interact synergistically with each other and induce serious diseases than to discuss which of these factors is the most virulent. A recent study showed that groupings by multi-locus sequence typing (MLST) using 7 housekeeping genes were associated with the prevalence of GC [60], although we reported a problem of this interpretation [61]. It may be better to classify *H. pylori* according to the structure of the bacteria instead of each virulence factor.

### Conclusion

*iceA1* was weakly, but significantly associated with PUD especially DU, whereas *iceA2* was inversely associated with PUD. A relationship between *iceA* and GC and GU was not found in this meta-analysis. It is possible that *iceA* is a discriminating factor for PUD, independent of *cagA*.

## Supporting Information

Figure S1 Flow diagram of study selection.
(TIF)

Figure S2 Results of the meta-analysis for the risk of duodenal ulcer in *iceA1*-positive *H. pylori* infections.
(TIF)

Figure S3 Results of the meta-analysis for the risk of gastric ulcer in *iceA1*-positive *H. pylori* infections.
(TIF)

Figure S4 Results of the meta-analysis for the risk of gastric cancer in *iceA1*-positive *H. pylori* infections.
(TIF)

Figure S5 Results of the meta-analysis for the risk of duodenal ulcer in *iceA2*-positive *H. pylori* infections.
(TIF)

Figure S6 Results of the meta-analysis for the risk of gastric ulcer in *iceA2*-positive *H. pylori* infections.
(TIF)

Figure S7 Results of the meta-analysis for the risk of gastric cancer in *iceA2*-positive *H. pylori* infections.
(TIF)

Table S1 Characteristics of studies included in the meta-analysis.
(XLS)
