# Supplementary figures and images for: Helicobacter pylori iceA, Clinical Outcomes, and Correlation with cagA: A Meta-Analysis
Source: PLoS One. 2012 Jan 18;7(1):e30354. doi: 10.1371/journal.pone.0030354 (PMC3261200; doi:10.1371/journal.pone.0030354)

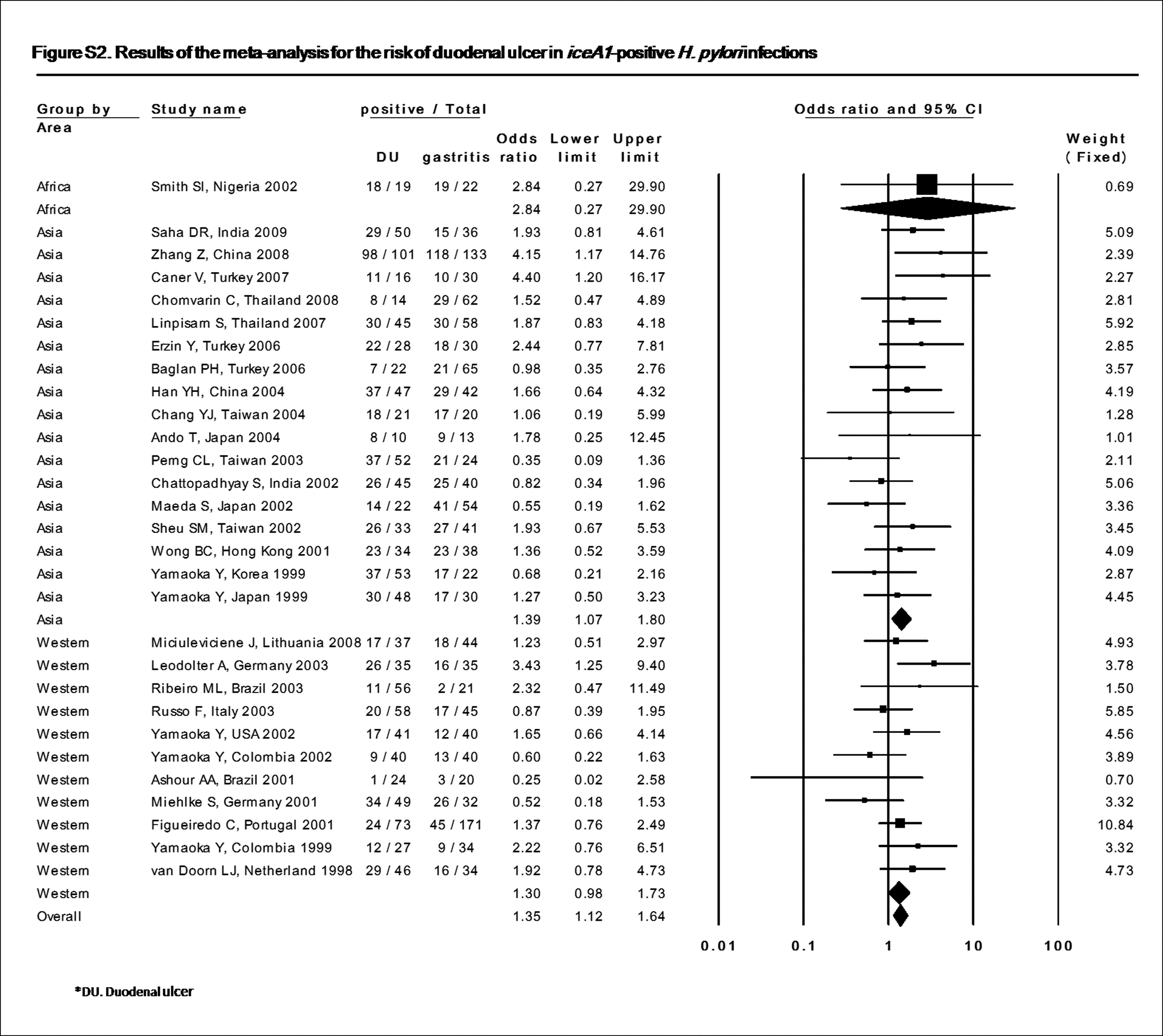

Supplement: Figure S2 — Results of the meta-analysis for the risk of duodenal ulcer in iceA1-positive H. pylori infections. (TIF) [file pone.0030354.s002.tif]

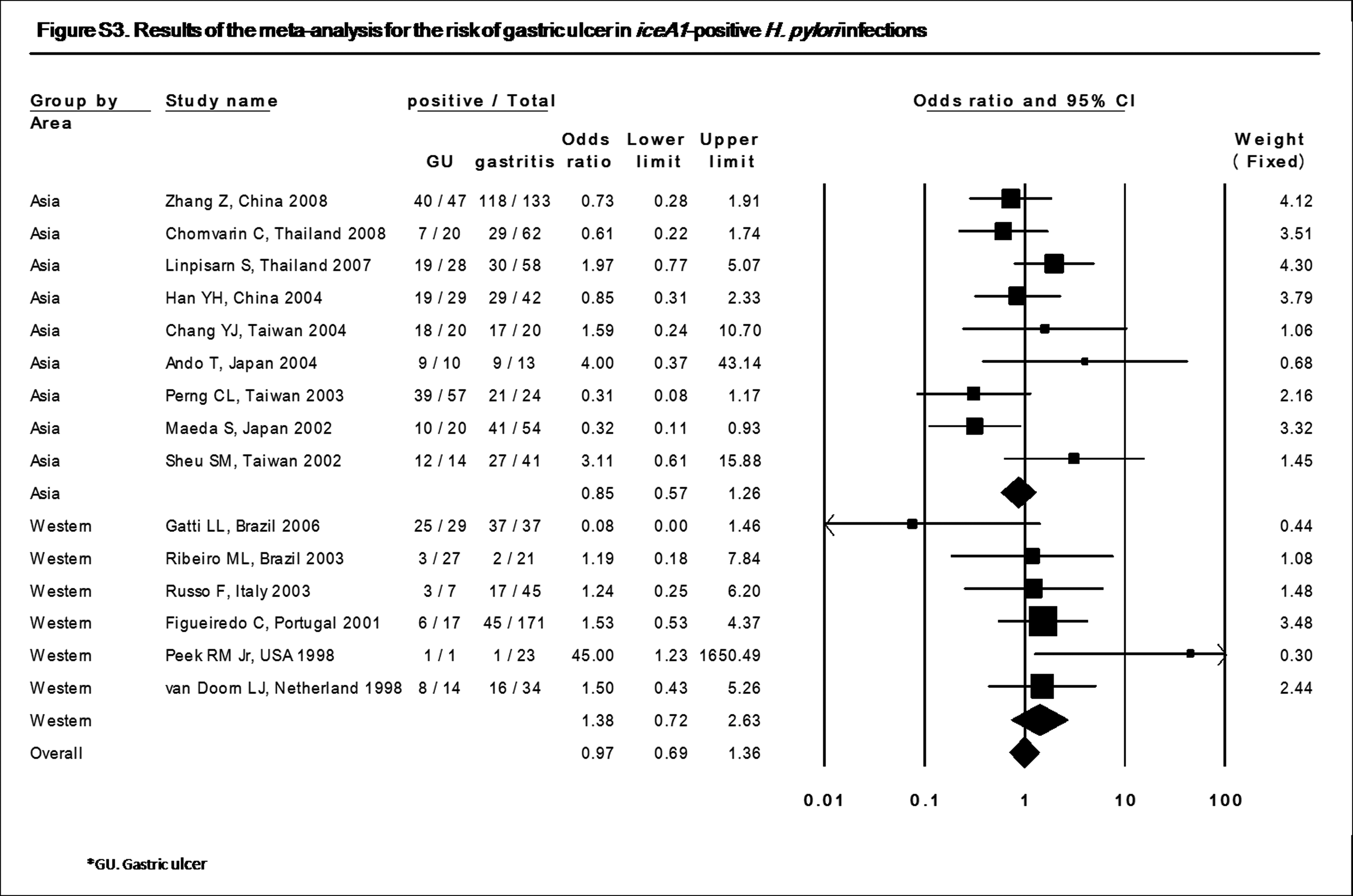

Supplement: Figure S3 — Results of the meta-analysis for the risk of gastric ulcer in iceA1-positive H. pylori infections. (TIF) [file pone.0030354.s003.tif]

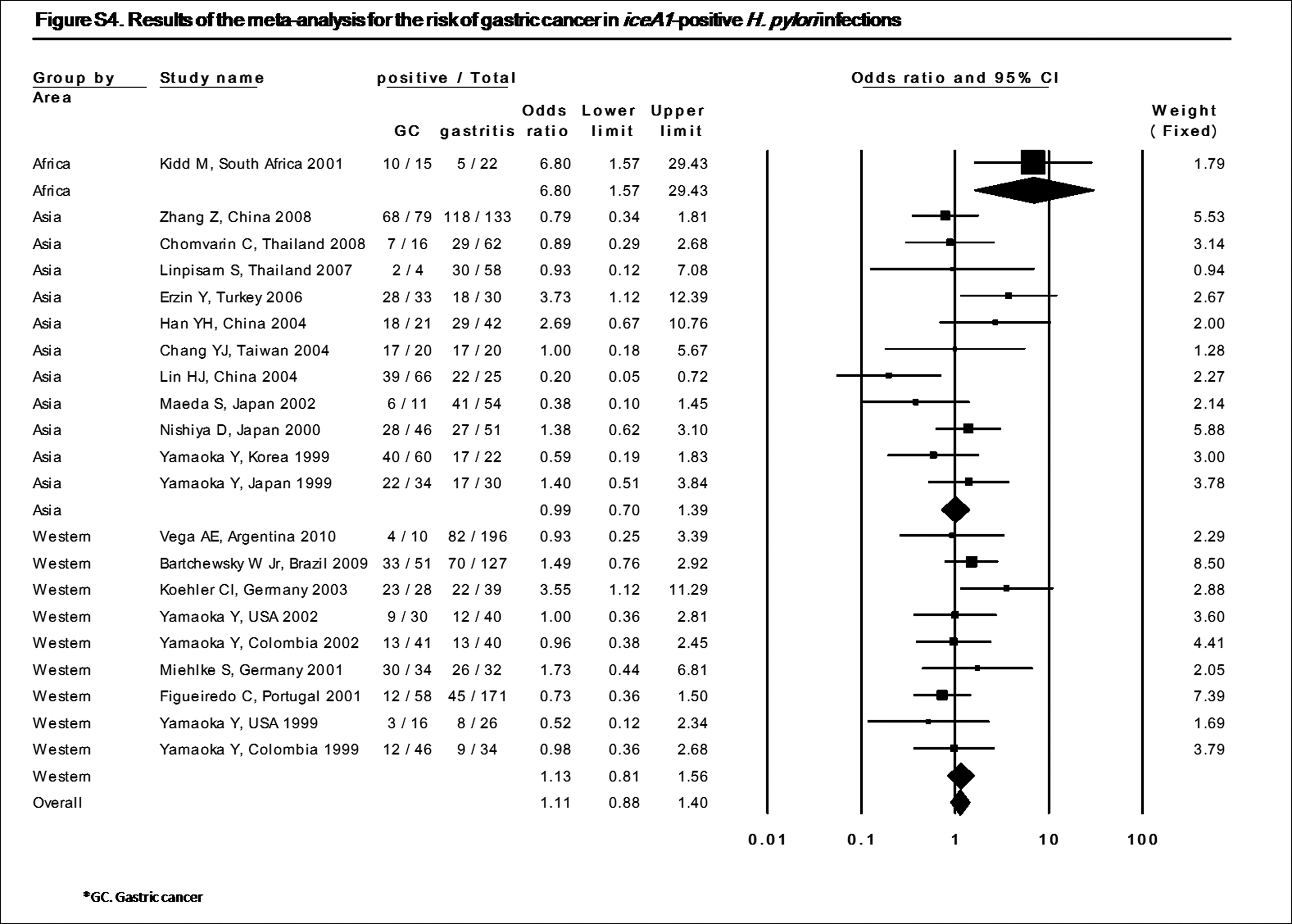

Supplement: Figure S4 — Results of the meta-analysis for the risk of gastric cancer in iceA1-positive H. pylori infections. (TIF) [file pone.0030354.s004.tif]

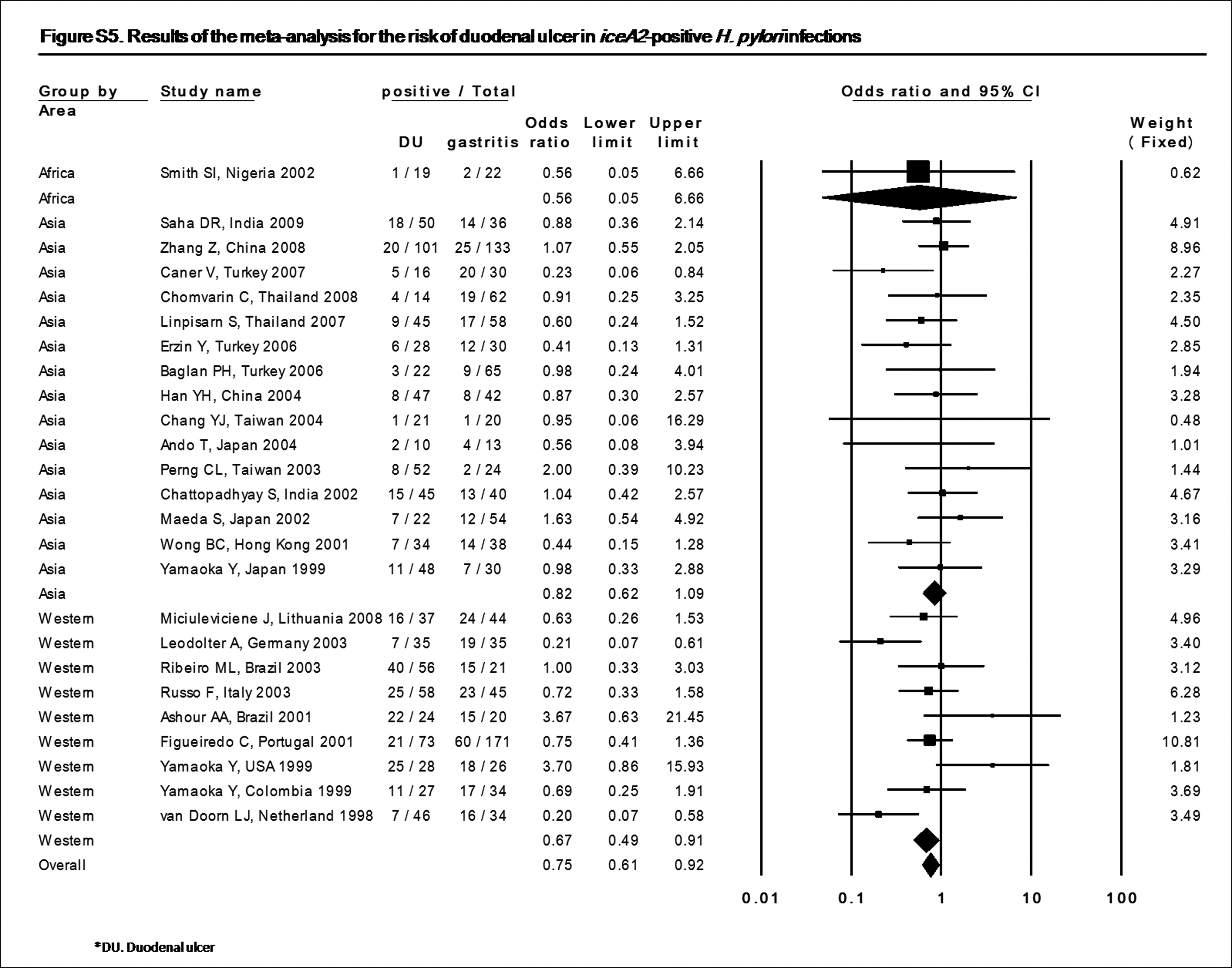

Supplement: Figure S5 — Results of the meta-analysis for the risk of duodenal ulcer in iceA2-positive H. pylori infections. (TIF) [file pone.0030354.s005.tif]

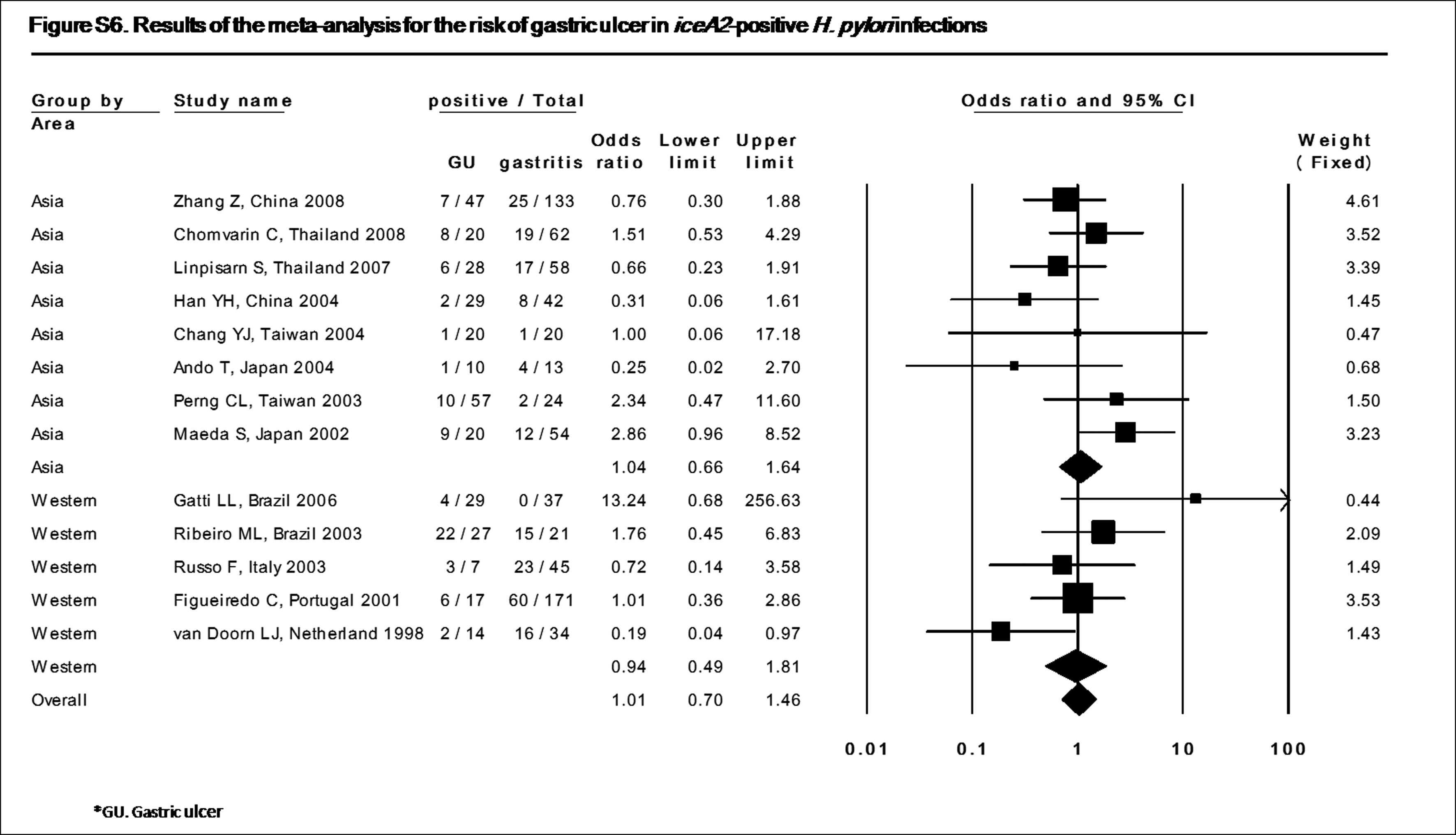

Supplement: Figure S6 — Results of the meta-analysis for the risk of gastric ulcer in iceA2-positive H. pylori infections. (TIF) [file pone.0030354.s006.tif]

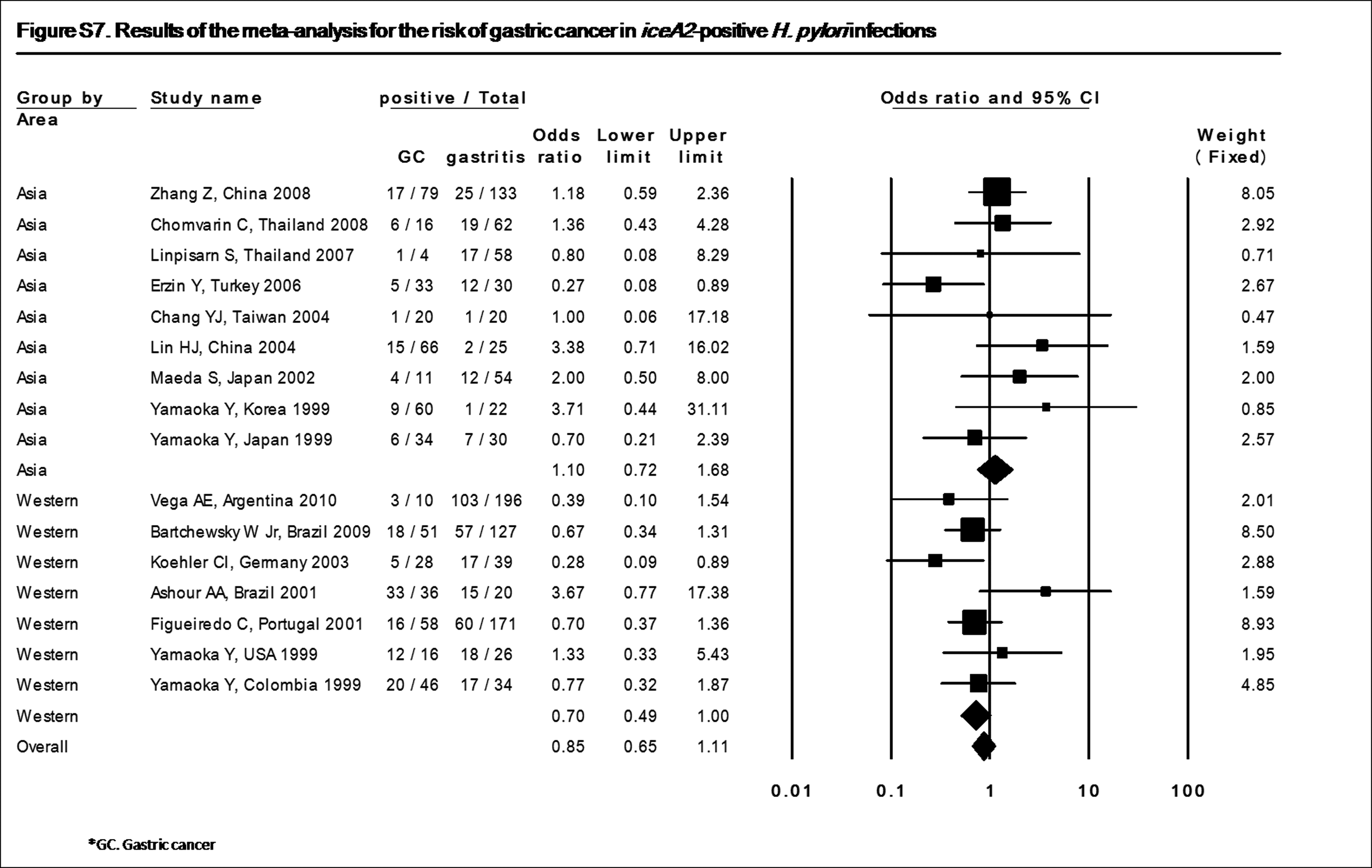

Supplement: Figure S7 — Results of the meta-analysis for the risk of gastric cancer in iceA2-positive H. pylori infections. (TIF) [file pone.0030354.s007.tif]
